# Word-based GWAS harnesses the rich potential of genomic data for *E. coli* quinolone resistance

**DOI:** 10.3389/fmicb.2023.1276332

**Published:** 2023-12-13

**Authors:** Negin Malekian, Srividhya Sainath, Ali Al-Fatlawi, Michael Schroeder

**Affiliations:** ^1^Biotechnology Center (BIOTEC), Technische Universität Dresden, Dresden, Germany; ^2^ITRDC, University of Kufa, Najaf, Iraq; ^3^Center for Scalable Data Analytics and Artificial Intelligence (ScaDS.AI), TU Dresden, Dresden, Germany

**Keywords:** genome-wide association studies (GWAS), microbial GWAS, word-based GWAS, unitig-GWAS, *E. coli*, quinolone, antibiotic resistance

## Abstract

Quinolone resistance presents a growing global health threat. We employed word-based GWAS to explore genomic data, aiming to enhance our understanding of this phenomenon. Unlike traditional variant-based GWAS analyses, this approach simultaneously captures multiple genomic factors, including single and interacting resistance mutations and genes. Analyzing a dataset of 92 genomic *E. coli* samples from a wastewater treatment plant in Dresden, we identified 54 DNA unitigs significantly associated with quinolone resistance. Remarkably, our analysis not only validated known mutations in *gyrA* and *parC* genes and the results of our variant-based GWAS but also revealed new (mutated) genes such as *mdfA*, the AcrEF-TolC multidrug efflux system, *ptrB*, and *hisI*, implicated in antibiotic resistance. Furthermore, our study identified joint mutations in 14 genes including the known *gyrA* gene, providing insights into potential synergistic effects contributing to quinolone resistance. These findings showcase the exceptional capabilities of word-based GWAS in unraveling the intricate genomic foundations of quinolone resistance.

## 1 Introduction

### 1.1 Conventional genome-wide association studies may not fully explore the potential of genomic data

While conventional GWAS methods have provided valuable insights, they may not fully explore the intricate genomic landscape underlying microbial phenotypes (Power et al., 2017), particularly in terms of variant interactions. These methods predominantly focus on individual single nucleotide polymorphisms (SNPs), potentially overlooking the significant influence of variant interactions and gene presence/absence on microbial phenotypic traits, including antibiotic resistance. To enhance our understanding and further complement conventional GWAS, embracing alternative strategies capable of simultaneously capturing the effects of genes and variants, whether they act individually or interactively, is crucial.

### 1.2 K-mers have the potential to broaden the horizons of conventional GWAS

K-mers, substrings of length k within biological sequences like DNA, RNA, or proteins, have the potential to broaden the horizons of conventional GWAS. By incorporating them into genome analysis, we can expand beyond the scope of conventional GWAS, which typically centers on single nucleotide polymorphisms (SNPs) or genes as the unit of study. Unlike these traditional approaches, k-mers offer the advantage of capturing the combined effects of both SNPs and genes simultaneously, providing a more comprehensive view of the genomic landscape underlying diseases and traits. Moreover, k-mers enable the capture of cumulative effects from multiple genomic variants in a single analysis, including rare and structural variants. K-mer-based GWAS stands out prominently in studying microbial genomes, as exemplified by Sheppard et al.'s work on *Campylobacter* isolates using 30-bp DNA sequences (Sheppard et al., 2013; Power et al., 2017).

### 1.3 K-mer-based GWAS pose challenges that can be mitigated by adopting unitigs

While k-mer-based GWAS excels in identifying genomic variants undetectable by variant-based GWAS, interpreting results proves challenging due to mapping difficulties and high redundancy. Unitigs, as unique non-redundant genome sequences, mitigate these challenges (Chaguza et al., 2020, 2022a). Longer than the common k-mer size, unitigs cover more extensive genomic regions, providing additional context for identified genomic variants. Mapping unitigs back to the original genome is also simplified compared to k-mers, as each unitig represents a unique non-redundant region. Previous studies support the efficacy of unitig-based GWAS approaches, offering specific genomic information and facilitating functional annotation of associated loci across various bacterial genomes, including *Mycobacterium* (Jaillard et al., 2018; Hang et al., 2019; Yano et al., 2021), *Staphylococcus* (Jaillard et al., 2018; Chaguza et al., 2022b; Raineri et al., 2022), and *E. coli* (Denamur et al., 2022; Van Wonterghem et al., 2022).

### 1.4 Unitig-based GWAS can study quinolone resistance

Unitig-based GWAS can be applied to any phenotype, including quinolone resistance. Quinolones are a broad-spectrum family of antibiotics used to treat both gram-negative and gram-positive bacterial infections (Emmerson and Jones, 2003). Quinolone resistance is a significant concern as it can lead to urinary tract and intraabdominal infections. Therefore, a better understanding of quinolone resistance mechanisms is necessary to develop effective approaches to overcome this issue.

### 1.5 Quinolone resistance mainly results from chromosomal mutations in *gyrA* and *parC*

Quinolones primarily target bacterial DNA topoisomerase II and topoisomerase IV. These enzymes are crucial for DNA replication, transcription, and recombination, as well as for helping to under- and over-wind DNA (Naeem et al., 2016). Quinolones aim to inhibit DNA synthesis and cell growth by targeting these enzymes and inhibiting their activity. Topoisomerase II consists of GyrA and GyrB subunits, while topoisomerase IV comprises ParC and two ParE subunits. GyrA is homologous to ParC, and GyrB is homologous to ParE (Hooper and Jacoby, 2015). While known point mutations in these genes play a significant role, other biomarkers can also impact quinolone resistance.

### 1.6 Known and new biomarkers from our previous variant-based GWAS serve as a baseline for this study

In our previous variant-based GWAS study of quinolone resistance (Malekian et al., 2021), we confirmed known mutations in *gyrA* and *parC*, and we also identified new mutations, mainly in *valS* and *bdcA* genes. Using these findings as our baseline, we explore the efficacy of unitig-based GWAS to discover additional single and joint resistance mutations for quinolone resistance using the same dataset.

### 1.7 Approach overview

**Figure 1:**
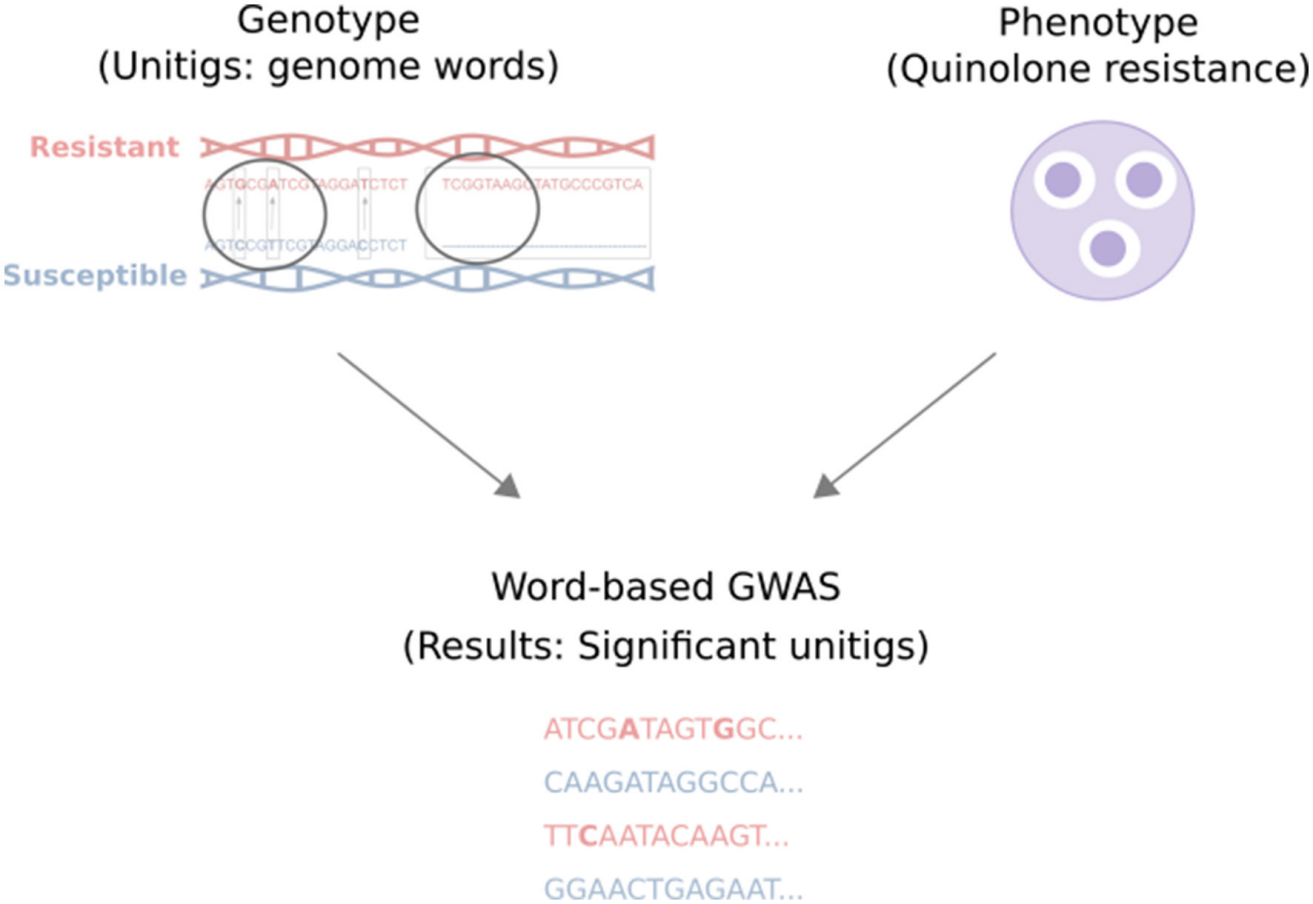
Overview of our study. The genotype and phenotype of wastewater *E. coli* samples were associated using a word-based GWAS analysis. Here, the genotype refers to the unitigs (genome words), and the phenotype refers to the samples' quinolone resistance value (the diameters of inhibition zones in the agar disk diffusion method). The analysis resulted in a list of significant unitigs that may contain single or joint candidate resistance mutations.

This study comprehensively analyzes 92 *Escherichia coli* (*E. coli*) genomes from a wastewater treatment plant in Dresden, Germany, and their corresponding quinolone resistance data. The approach includes extracting unitigs from the genomic data of the samples, applying quality control measures, and investigating their association with quinolone resistance labels for levofloxacin, norfloxacin, ciprofloxacin, and nalidixic acid. Subsequently, the unitigs are mapped back to a reference genome to determine the genes and mutations they encompass. Finally, the identified genes and mutations are investigated for their potential role in conferring resistance. An overview of our study is shown in Figure 1.

## 2 Methods

### 2.1 Sequence data and resistance phenotype

The dataset comprised 92 *E. coli* genomes from a municipal wastewater treatment plant in Dresden, Germany, and their antibiotic resistance values (i.e., the diameters of inhibition zones in the agar disk diffusion method) against 20 widely prescribed antibiotics, including four quinolones (levofloxacin, norfloxacin, ciprofloxacin, and nalidixic acid). The genomic data can be accessed directly from NCBI's assembly database under the project identifier PRJNA380388 (https://www.ncbi.nlm.nih.gov/bioproject/PRJNA380388/). Antibiotic resistance data is available within the Biosample field of the same dataset. For further details, (see Mahfouz et al., 2018).

### 2.2 Identifying unitigs

To identify unitigs, we used the unitig-counter v1.1.0 software tool (Lees et al., 2020). The input for the unitig-counter was the genomic assembly of all our isolates in the FASTA format. The algorithm of this tool involves constructing a compressed de Bruijn graph from all the input genome sequences and identifying contiguous sequences of nucleotides, known as unitigs, from the resulting graph.

### 2.3 Association analysis

We used the fixed-effect generalized linear model (SEER) of the Pyseer tool v1.3.9 (Lees et al., 2018) to associate the presence/absence of unitigs with continuous antibiotic resistance values, similar to the approach used in our previous variant-based GWAS (Malekian et al., 2021). Population structure was controlled by adding covariates to the linear regression model. We utilized multidimensional scaling (MDS) on distances from the phylogenetic tree constructed from the VCF file using VCF-kit v0.1.6 (Cook and Andersen, 2017). Selected components from the MDS model, the number of components at the knee of a scree plot, served as covariates to control for population structure. Significant unitigs were retained after applying the Bonferroni correction. To calculate the Bonferroni-corrected threshold, we used the Pyseer tool, which divides the standard significance level of 0.05 by the number of distinct unitig patterns, resulting in a p-value threshold of 8.06 × 10^−8^.

### 2.4 Unitig annotation

We mapped significant unitigs to our reference genome (*E. coli* K-12 MG1665, accession NC_000913.3) using BWA v0.7.17-r1188 (Li, 2013) and performed variant calling with Samtools v1.7 and Bcftools v1.8 (Danecek et al., 2021). The variants were annotated using SnpEff v5.1 (Cingolani et al., 2012). For variant-free unitigs associated with antibiotic susceptibility instead of resistance, we searched for resistance mutations in the complementing isolate set. The complementing isolate set comprises unitigs covering the same genomic regions and associated with antibiotic resistance (*p*-value < 0.05). Our final set of resistance mutations included mutations in both the mutated unitigs and the complementing isolate set of unmutated unitigs.

### 2.5 Functional analysis of annotated unitigs

We investigated the function of genes and variants mapped to our significant unitigs using the UniProt knowledgebase (The UniProt Consortium, 2023) and the EcoCyc *E. coli* database (Keseler et al., 2010).

### 2.6 Network analysis of new genes

To investigate connections among new (mutated) genes not captured in our previous variant-based GWAS, we employed STRING-db (Szklarczyk et al., 2015). Default settings were utilized, which incorporate multiple sources of evidence such as text mining, experimental data, databases, co-expression, neighborhood, gene fusion, and co-occurrence. The edge thickness setting was adjusted to indicate the strength of data support.

### 2.7 Antibiotic resistance analysis of unitigs

To assess the association between the genes containing significant unitigs and antibiotic resistance, we employed the CARD database (McArthur et al., 2013) and relevant literature.

## 3 Results and discussion

This study examined correlations between unitigs and antibiotic resistance labels for four quinolones. The first objective was to validate the coverage of the positive controls, which consisted of known mutations in *gyrA* and *parC*, by the unitigs. The word-based GWAS was also expected to confirm previously identified mutations from the variant-based GWAS, particularly the mutations in the *bdcA* and *valS* genes. Moreover, the study aimed to explore the possibility of discovering new mutations or genes that the variant-based GWAS had not detected. Finally, but most importantly, the investigation aimed to uncover joint mutations that could potentially interact with each other.

### 3.1 A total of 54 highly-quality significant unitigs were identified

We extracted a total of 1,491,067 unitigs from our genomes. Initially, we excluded unitigs that appeared in over 99% of the dataset, resulting in a reduction to 1,209,817 unitigs, representing a 19% decrease. We then associated the presence/absence of these unitigs with resistance values for the four quinolones: levofloxacin, norfloxacin, ciprofloxacin, and nalidixic acid. Applying a Bonferroni-corrected threshold (*p*-value: 8.06 × 10^−08^), we identified 100 highly significant unitigs: 33 for levofloxacin, 65 for norfloxacin, 17 for ciprofloxacin, and 1 for nalidixic acid. Some unitigs were significant for more than one antibiotic, resulting in a total count that differs from 100.

To understand the biological significance of the unitigs, we mapped them to the reference genome (*E. coli* K-12 substr. MG1655) to determine the associated genes and variants. Among the 100 highly significant unitigs, 54 possessed clear and high-quality annotations, constituting our final set of significant unitigs: 18 for levofloxacin, 36 for norfloxacin, 12 for ciprofloxacin, and 1 for nalidixic acid (see Supplementary material).

The 54 significant unitigs captured both some of the previously identified mutations and novel mutations in unexplored genes. Additionally, we observed joint mutations within unitigs, and their combinations exhibited significant correlations with quinolone resistance. A summary of our findings is presented in Table 1, indicating the potential utility of word-based GWAS in identifying genomic mutations that may be associated with antibiotic resistance. We will elaborate on each of our new findings in the following.

**Table 1:** Summary results of word-based GWAS for quinolone resistance.

**Description**	**Levofloxacin**	**Norfloxacin**	**Ciprofloxacin**	**Nalidixic acid**
Known mutations	*parC* S80I*gyrA* S83L	*parC* S80I*parE* L416F*gyrA* S83L*gyrA* D87N	*parC* S80I*gyrA* S83L*gyrA* D87N	*gyrA* S83L
Mutations identifiedby variant-based GWAS	*valS* R733	*bdcA* G135S*valS* R733		
New (mutated) genes	*cheAflhBcheZstfRyecFgnd*	*nrdDyiclyicHacrFyhdVacrEgspAymfLlldPsgbUyiaMyiaNaldBmutMdutptrBmdfA*	*hisIstfRymfL*	
Genes withjoint mutations	*cheAcheZstfRvalSgnd*	*gyrAnrdDvalSyicHymfLlldPyiaNyiaMaldBdut*	*gyrAstfR*	

Using word-based GWAS, we identified known mutations as well as some of the mutations previously discovered through our variant-based GWAS. Additionally, we found new mutations in previously unexplored genes. We also observed joint mutations within unitigs, and their combinations were significantly correlated with quinolone resistance.

### 3.2 Significant unitigs captured known mutations

By mapping our genomes to the reference, *E. coli* K-12 substr. MG1655, we successfully identified significant unitigs harboring known biomarkers of quinolone resistance. Notably, these unitigs encompassed mutations in key genes such as *gyrA* and *parC*. For levofloxacin resistance, we observed mutations including *parC* S80I and *gyrA* S83L. Similarly, for norfloxacin resistance, we found *parC* S80I, *parE* L416F, *gyrA* S83L, and *gyrA* D87N. In the case of ciprofloxacin resistance, the identified known mutations were *parC* S80I, *gyrA* S83L, and *gyrA* D87N. Additionally, *gyrA* S83L was associated with resistance to nalidixic acid. Detailed information about these unitigs, including p-values, effect sizes, frequencies, and more, can be found in Supplementary material.

Additionally, our top significant unitigs were located within the quinolone resistance-determining regions (QRDRs) of GyrA. The QRDRs are amino acids between 67 and 106, which are conserved regions involved in DNA binding and are well-known to cause quinolone resistance when mutations occur (Varughese et al., 2018). The QRDR is near tyrosine 122, which binds to DNA during DNA double-strand breaks or DNA single-strand rejoins. Non-synonymous mutations (*gyrA* S83L, *gyrA* D87N) and synonymous mutations (*gyrA* R91R, *gyrA* V85V) were observed in this region. Among the unitigs in this region, those without any mutations were correlated with antibiotic susceptibility, while those with mutations were correlated with antibiotic resistance.

### 3.3 Significant unitigs captured some of the mutations previously identified in our variant-based GWAS study

Our previous variant-based GWAS analysis (Malekian et al., 2021) discovered significant correlations between specific variants in the *valS* and *bdcA* genes, which are involved in translation and biofilm formation, respectively, and quinolone resistance. Building upon this finding, our subsequent GWAS analysis at the unitig level unveiled additional insights. Specifically, we found that two unitigs within the *valS* gene were strongly associated with resistance to both levofloxacin and norfloxacin, while one unitig within the *bdcA* gene exhibited a significant correlation with norfloxacin susceptibility. These results further reinforce the importance of these genomic regions in developing quinolone resistance.

Within the unitigs associated with the *valS* gene, one unitig contained two synonymous mutations, namely *valS* R733R and *valS* A730A. Notably, the variant *valS* R733R had also been identified in our previous variant-based GWAS analysis. In contrast, the other unitig related to *valS* did not exhibit any variants, and thus, it displayed a correlation with antibiotic susceptibility rather than resistance. Similarly, the unitig linked to the *bdcA* gene did not include any variants and was associated with antibiotic susceptibility. It is intriguing to observe that this unitig encompassed the region harboring the G135S variant previously identified in *bdcA* among resistant isolates. These findings highlight the potential of word-based GWAS in identifying potential antibiotic resistance targets, expanding our understanding of the genomic landscape of quinolone resistance. For more comprehensive information on the significant unitigs in the *bdcA* and *valS* genes, including p-values, effect sizes, frequencies, and other details, please refer to Supplementary material.

### 3.4 Significant unitigs captured new mutations

Additionally, the word-based GWAS revealed mutations in new genes linked to quinolone resistance. The list of these new (mutated) genes can be found in Table 1. For more in-depth information regarding the unitigs containing these mutations, including p-values, effect sizes, frequencies, and other relevant details (see Supplementary material).

Some of these new (mutated) genes, such as *mdfA*, the AcrEF-TolC multidrug efflux system, *ptrB*, and *hisI* have been previously associated with antibiotic or quinolone resistance in the literature.

The *mdfA* gene encodes a multidrug efflux pump that, when upregulated, is strongly linked to resistance to several antibiotics, including quinolones (Yasufuku et al., 2011; Gu et al., 2021; Li and Ge, 2023). In this study, we found a region within the *mdfA* gene that displayed a strong association with norfloxacin susceptibility. This region was mostly unmutated in susceptible samples but mutated in resistant samples, with a synonymous variant, A42A.

The AcrEF-TolC multidrug efflux system is a homolog of the well-known AcrAB-TolC multidrug efflux system and is composed of three genes, *acrF, acrE*, and *tolC*. Prior research has demonstrated that overexpression of the *acrF* and *acrE* genes results in antibiotic resistance, including fluoroquinolones (Ma et al., 1993; Okusu et al., 1996; Lau and Zgurskaya, 2005). Our study found one unitig in the *acrE* gene and one unitig in the *acrF* gene strongly associated with norfloxacin susceptibility.

Overexpression of the *hisI* and *ptrB* genes has been shown to increase ciprofloxacin resistance in *Escherichia coli* (Ranjith et al., 2017) and *Pseudomonas aeruginosa* (Sun et al., 2014), respectively. This study found a non-synonymous mutation, L46I, and a synonymous mutation, T52T, in the *hisI* gene correlated with ciprofloxacin resistance. However, for the *ptrB* gene, we found a non-synonymous mutation, V629I, was linked to norfloxacin resistance, which is in the relative vicinity of the predicted active sites at positions 617 and 652; refer to UniProt ID P24555.

Furthermore, while the direct association of the remaining new (mutated) genes with antibiotic resistance was not explored in the literature, we identified evidence indicating potential links for 19 out of the 25 new (mutated) genes (refer to Table 2). As the table shows, these genes are actively involved in crucial functions such as multidrug efflux, bacterial tolerance, bacterial persistence, fitness to stress, and biofilm formation. Thus, they present plausible candidates that could potentially be linked to antibiotic resistance.

**Table 2:** The (potential) role of new (mutated) genes in antibiotic resistance (AR).

**Gene(s)**	**(Potential) role in AR**	**Description**
*mdfA*	Multidrug efflux	Overexpression of the gene confers broad-spectrum antibiotic resistance, including resistance to fluoroquinolones, by actively pumping out antibiotics (Edgar and Bibi, 1997; Alcock et al., 2020).
*acrE, acrF, yhdV*	Multidrug efflux	The *acrF* and *acrE* genes are part of the AcrEF-TolC multidrug efflux system, and its overexpression is associated with multidrug resistance (Okusu et al., 1996; Alcock et al., 2020). The *yhdV* gene is in the same operon as *acrF* gene.
*ptrB*	Bacterial tolerance	Mutation in this gene enhances bacterial tolerance to stresses like ciprofloxacin by reducing pyocin production (Sun et al., 2014). Pyocins are bacteriocins, proteins bacteria produce to combat similar bacteria.
*aldB*	Bacterial persistence	The gene knockdown decreased *E. coli* persistence under some conditions (Kawai et al., 2018). Persistence refers to the ability of bacteria to survive lethal doses of antibiotics without genetic mutations.
*yicI, yicH*	Fitness to stress	These genes can improve the overall fitness of bacteria under stress conditions, but their role in antibiotic resistance is not explored yet (Répérant et al., 2011).
*cheA, cheZ, flhB*	Biofilm formation	Part of the *flhAB cheZYBR tap tarc cheWA motBA flhCD* gene cluster, which is involved in chemotaxis and biofilm formation (Tirumalai et al., 2019).
*ymfL, stfR*	Biofilm formation	The *ymfL* gene is within the prophage element e14, while the *stfR* gene is within the prophage element rac. Both e14 and rac phage remnants affect biofilm formation, as removing them from *E. coli* K-12 impairs biofilm production (Mehta et al., 2004; Fortier and Sekulovic, 2013).
*yecF*	Biofilm formation	Mutation in the *sdiA* gene, which belongs to the same operon as *yecF*, helps form thicker biofilm and higher motility than the wild type and complemented strains (Culler et al., 2018).
*hisI*	Biofilm formation	The upregulation of this gene, involved in amino-acid and metabolite transport, along with other genes, likely contributes to antibiotic resistance in *E. coli* biofilms (Ranjith et al., 2017).
*nrdD*	Biofilm formation	This gene, which is essential for DNA synthesis and repair, was upregulated after prolonged exposure to biocides that caused biofilm development and inhibited motility (Merchel Piovesan Pereira et al., 2020).
*gpsA*	Biofilm formation	The gene's role in antibiotic resistance remains unclear but has been studied in biofilm formation. Mutants lacking this gene showed a significant negative impact on biofilm formation (Qin et al., 2019).
*yiaM, yiaN*	Biofilm formation	These genes belong to the *yiaMNO* gene cluster. Deleting the *yiaMNO* genes in *E. coli* led to significant alterations in its growth pattern, ability to survive in high-salt conditions, and the formation of biofilms (Plantinga et al., 2005).

We found a potential link to AR for 19 new (mutated) genes out of 25, presented here.

**Figure 2:**
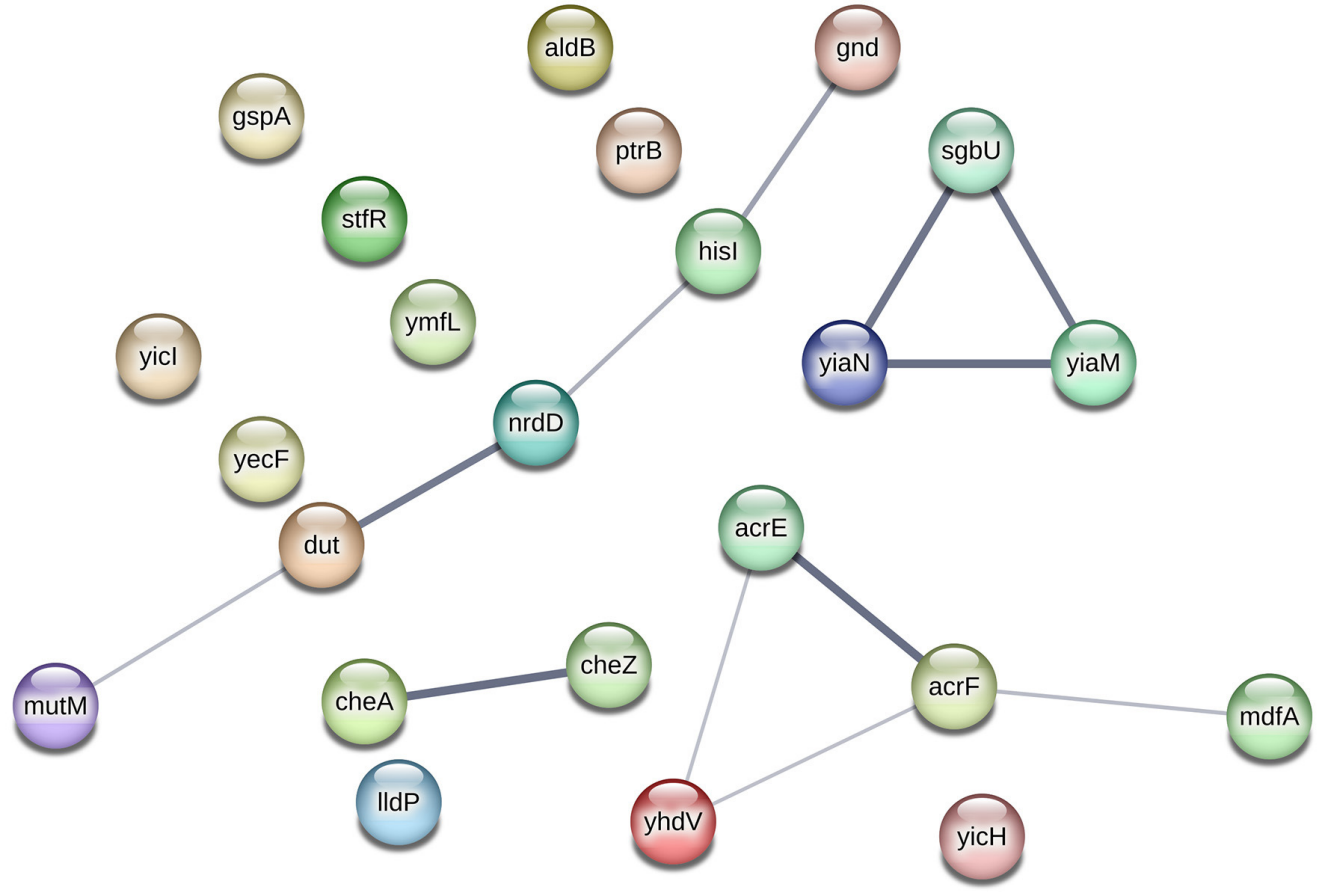
Network analysis of new (mutated) genes discovered by word-based GWAS, using STRING database (Szklarczyk et al., 2015). The edge thickness indicates the strength of data support. The network analysis shows evidence of interactions between four groups of genes: (1) the *gnd, hisI, nrdD, dut*, and *mutM* genes, (2) the *cheA* and *cheZ* genes, (3) *sgbU, yiaN*, and yiaM, and (4) the *acrE, yhdV, acrF*, and *mdfA* genes. However, there is no evidence of interactions among other genes.

Moreover, the involvement of new (mutated) genes in antibiotic resistance through interactions with other genes is plausible, as demonstrated in Figure 2. The interaction network in this figure shows the potential collaborative relationships among these genes. For instance, genes like *gnd, dut*, and *mutM*, which lacked evidence of a direct link to antibiotic resistance, might still confer resistance when cooperating with the *hisI* and *nrdD* genes, possibly aiding in biofilm formation. Similarly, the *sgbU* gene could contribute to antibiotic resistance in partnership with the *yiaM* and *yiaN* genes, potentially through biofilm formation mechanisms.

### 3.5 Significant unitigs captured joint mutations

Significant unitigs captured joint mutations that could interact with each other to drive quinolone resistance. Unlike conventional variant-based GWAS, which only considers individual mutations, word-based GWAS analyzes larger genome portions, enabling the detection of mutation interactions. The word-based GWAS identified highly significant unitigs for quinolone resistance that contain multiple variants, suggesting that the interaction between these mutations contributes to the development of antibiotic resistance. Some of these variants were found in well-known targets of quinolone resistance, such as *gyrA* and *parC*, while others were located in new genes, including *galF, cheA, yiaM*, and *cheZ*. Notably, these variations were in crucial positions, such as the quinolone-resistance determining regions for *gyrA* (Varughese et al., 2018), the substrate binding site neighborhood for *gnd* (UniProt ID P00350), or an essential catalytic domain for the *cheA* (UniProt ID P07363), *yiaM* (UniProt ID P37674), and *cheZ* (UniProt ID P0A9H9) genes. For a full list of genes that contain joint mutations, refer to Table 1. For further details on unitigs that contain such mutations, such as *p*-value, effect size, frequency, etc., (see Supplementary material).

### 3.6 General discussion

#### 3.6.1 Word-based GWAS using unitigs yielded significant quinolone resistance findings

The word-based GWAS using unitigs was highly effective for analyzing quinolone resistance. Comparing it to k-mer level analyses (results not provided here), we found unitig analysis superior in terms of interoperability and significant findings. We identified 54 unitigs containing regions covering known mutations in *gyrA* and *parC* genes, as well as previously identified mutations using our variant-based GWAS in *bdcA* and *valS*.

Additionally, we discovered new variants in previously unexplored genes, some of which have been linked to antibiotic resistance. However, further investigation is required to confirm these associations. Notably, these new genes were missed by both our previous variant-based GWAS (Malekian et al., 2021) and the positive selection (Malekian et al., 2022) analysis of *E. coli* for antibiotic resistance. Therefore, our unitig-based GWAS allowed for a comprehensive analysis of quinolone resistance, showcasing the value of this approach in identifying genomic factors associated with antibiotic resistance.

#### 3.6.2 Word-based and variant-based GWAS do not fully overlap in their detection of single mutations

Word-based GWAS and variant-based GWAS did not identify exactly the same list of single mutations. The reason behind this relies on the way that unitigs are built. In the context of variant-based GWAS, cases consist of isolates harboring particular mutations, while controls encompass isolates devoid of these mutations. Conversely, in word-based GWAS, the landscape is more complex, encompassing unitigs that contain a specific single mutation, unitigs devoid of the mutation, and unitigs with the specific mutation alongside other mutations. Consequently, when focusing on the detection of single mutations, the results from variant-based GWAS are considered more reliable.

#### 3.6.3 A significant portion of the identified resistance mutations are synonymous

The list of significant resistant mutations contain many synonymous mutations. Unlike non-synonymous mutations that directly impact the protein product, structure, and function, synonymous mutations exert their influence indirectly by affecting processes such as splicing, RNA stability, RNA folding, translation, and cotranslational protein folding (Sharma et al., 2019). As a result, these synonymous mutations play an indirect yet vital role in shaping the phenotype of interest.

#### 3.6.4 Word-based GWAS suggests specific mutations and their interactions drive quinolone resistance

Our study emphasizes the significance of mutations and their interactions over individual resistance gene presence in quinolone resistance. However, the factors influencing resistance may vary depending on the antibiotic under study. We detected joint variants in the known target *gyrA* and new genes (*galF, cheA, yiaM*, and *cheZ*), all situated in critical regions. These mutations occupy essential positions, such as quinolone-resistance determining regions for *gyrA*, substrate binding site neighborhood for *gnd*, and catalytic domains for *cheA, yiaM*, and *cheZ*.

#### 3.6.5 While word-based GWAS demonstrates computational power, biological experimental validation remains essential to confirm findings

We conducted our analysis on an *E. coli* dataset of 92 samples from a wastewater treatment plant, which might initially appear modest in size. However, the dataset's capability to affirm the presence of positive controls, notably the *gyrA* and *parC* genes, instilled confidence in its reliability for conducting thorough statistical analyses and evaluating the efficacy of the word-based GWAS approach. This robust dataset, anchored in unbiased sequencing and antibiotic resistance measurement methods (Mahfouz et al., 2018), underscores the comprehensiveness of our study. Word-based GWAS in bacterial studies can unveil genomic sequence patterns associated with bacterial phenotypes beyond single-nucleotide variations, providing efficient phenotype predictions and valuable functional insights. However, experimental validation is necessary to affirm the biological findings.

## 4 Conclusion

This study utilized a word-based GWAS to overcome the limitations of conventional variant-based GWAS analyses for genomic data. By examining genome words in 92 wastewater *E. coli* genomes, the study identified 54 significant words strongly associated with quinolone resistance. Positive controls, including known mutations in *gyrA* and *parC*, were validated, along with previously identified mutations in *bdcA* and *valS* from variant-based GWAS. Additionally, novel (mutated) genes such as *mdfA*, the acrEF-TolC multidrug efflux system, *ptrB*, and *hisI* were discovered, which are known to contribute to antibiotic resistance. Notably, the study revealed potentially interacting mutations in 14 genes, one of them being the well-known quinolone target *gyrA*. These mutations are located in critical sites, including quinolone-resistance determining regions in *gyrA*, the neighborhood of the substrate binding site in *gnd*, and the catalytic domains of *cheA, yiaM*, and *cheZ*. This finding suggests that quinolone resistance may not only result from individual mutations identified by variant-based GWAS but also from potential interactions between mutations.

## Data availability statement

Publicly available datasets were analyzed in this study. This data can be found here: https://www.ncbi.nlm.nih.gov/bioproject/PRJNA380388/.

## Author contributions

NM: Conceptualization, Data curation, Formal analysis, Investigation, Methodology, Validation, Visualization, Writing—original draft, Writing—review & editing. SS: Data curation, Formal analysis, Investigation, Validation, Writing—original draft, Writing—review & editing. AA-F: Writing—review & editing, Formal analysis. MS: Formal analysis, Writing—review & editing, Project administration, Supervision.
