# Supplementary material for: Word-based GWAS harnesses the rich potential of genomic data for E. coli quinolone resistance
Source: Front Microbiol. 2023 Dec 13;14:1276332. doi: 10.3389/fmicb.2023.1276332 (PMC10751334; doi:10.3389/fmicb.2023.1276332)
Supplement: Supplementary file 1 [file Data_Sheet_1.pdf]

Supplementary Material

Supplementary Table 1: Unitigs that significantly correlate with **levofloxacin** resistance (Bonferroni-corrected p-value threshold: 8.06×10^{-8}). The effect field shows any mutations in the unitigs that could have contributed to the resistance. If there is a C superscript in this field, it means the mutations occurred in the complementary set of isolates without this unitig, indicating a correlation with antibiotic susceptibility instead of resistance, as reflected by the sign of the effect size (beta). An E superscript in the effect field denotes the effect size for the extension of the unitig when the initial mapping attempt was unsuccessful. The frequency (freq.) field shows the relative frequency among isolates, while beta denotes the effect size, and SE denotes the standard error of the fit on beta.

Position	Length	Gene	Effect	Freq.	Beta	SE	P-value
3165735-3165765	31	*parC*	S80I^{C}	0.92	1.68	0.23	1.83×10^{-10}
3165705-3165765	61	*parC*	S80I	0.05	-1.75	0.26	2.65×10^{-09}
2339167-2339203	37	*gyrA*	S83L	0.13	-1.25	0.18	8.85×10^{-10}
1974095-1974155	61	*cheA*	L405L<br>V400V	0.03	-2.22	0.33	1.87×10^{-09}
1974012-1974058	47	*cheA*	E428E	0.15	-2.20	0.33	2.85×10^{-09}
1965376-1965436	61	*flhB*	A262A	0.02	-2.64	0.41	5.01×10^{-09}
1973981-1974041	61	*cheA*	T440T<br>L439L<br>G438G	0.02	-2.64	0.41	5.01×10^{-09}
1974284-1974344	61	*cheA*	P337P<br>R335R<br>S334S<br>V332V	0.02	-2.64	0.41	5.01×10^{-09}
1966570-1966624	55	*cheZ*	V148V<br>G146G	0.02	-2.64	0.41	5.01×10^{-09}
1974029-1974067	39	*cheA*	T419T^{E}	0.14	-1.76	0.30	8.03×10^{-08}
1429889-1429944	56	*stfR*	S293S	0.08	-1.43	0.23	2.58×10^{-08}
1429777-1429893	117	*stfR*	E244E<br>A253A<br>S254S<br>S255T<br>S258S<br>A261A<br>A271V	0.07	-1.52	0.25	2.92×10^{-08}
1429867-1429918	52	*stfR*	N280N<br>G289E	0.07	-1.52	0.25	2.92×10^{-08}
4481616-4481669	54	*valS*	R733R<br>A730A	0.07	-1.55	0.26	6.72×10^{-08}
4481616-4481647	32	*valS*	R733R^{C}<br>A730A^{C}	0.92	1.54	0.26	7.92×10^{-08}
1995877-1995937	61	*yecF*	L30L	0.02	-2.64	0.41	5.01×10^{-09}
1995877-1995915	39	*yecF*	L30L^{C}	0.98	2.64	0.41	5.01×10^{-09}
2100348-2100393	46	*gnd*	L303L<br>V302V	0.02	-2.64	0.41	5.01×10^{-09}

Supplementary Table 2: Unitigs that significantly correlate with **norfloxacin** resistance (Bonferroni-corrected p-value threshold: 8.06×10^{-8}). The effect field shows any mutations in the unitigs that could have contributed to the resistance. If there is a *C* superscript in this field, it means the mutations occurred in the complementary set of isolates without this unitig, indicating a correlation with antibiotic susceptibility instead of resistance, as reflected by the sign of the effect size (beta). The frequency (freq.) field shows the relative frequency among isolates, while beta denotes the effect size, and SE denotes the standard error of the fit on beta.

Position	Length	Gene	Effect	Freq.	Beta	SE	P-value
3165735-3165765	31	*parC*	$S80I^C$	0.92	2.56	0.25	7.67×10^{-17}
3165705-3165765	61	*parC*	S80I	0.05	-2.50	0.30	1.46×10^{-12}
3165724-3165761	38	*parC*	$S80I^C$	0.78	2.03	0.25	1.55×10^{-12}
3165705-3165753	49	*parC*	$S80I^C$	0.77	1.93	0.24	2.01×10^{-12}
3165732-3165764	33	*parC*	$S80I^C$	0.79	1.76	0.25	3.86×10^{-10}
3174121-3174181	61	*parE*	L416F	0.03	-2.68	0.44	3.14×10^{-08}
2339167-2339203	37	*gyrA*	S83L	0.13	-1.69	0.22	1.16×10^{-11}
2339167-2339203	37	*gyrA*	$S83L^C$	0.86	1.52	0.21	3.26×10^{-10}
2339132-2339192	61	*gyrA*	R91R<br>D87N<br>V85V<br>S83L	0.04	-2.50	0.36	5.32×10^{-10}
4473625-4473661	37	*bdcA*	$G135S^C$	0.78	1.80	0.27	4.21×10^{-09}
4461917-4461949	33	*nrdD*	$N251S^C$<br>$R244H^C$<br>$T237T^C$	0.96	2.65	0.44	4.02×10^{-08}
4481616-4481669	54	*valS*	A730A<br>R733R	0.07	-1.94	0.33	6.74×10^{-08}
4481616-4481647	32	*valS*	$R733R^C$<br>$A730A^C$	0.92	1.93	0.33	7.48×10^{-08}
3832620-3832657	38	*yicI*	L630L	0.04	-2.39	0.38	1.10×10^{-08}
3832654-3832701	48	*yicI*	A617A				
3831595-3831629	35	*yicH*	$G358G^C$<br>$G377G^C$<br>$S381S^C$	0.96	2.66	0.43	2.87×10^{-08}
3832633-3832675	35	*yicI*	A623S	0.03	-2.68	0.44	3.14×10^{-08}
3417549-3417579	31	*acrF*	$Q229Q^C$	0.67	2.13	0.34	1.24×10^{-08}
3418362-3418400	39	*yhdV*		0.96	2.66	0.43	2.87×10^{-08}
3413900-3413958	59	*acrE*		0.96	2.65	0.44	4.02×10^{-08}

Table continued from the previous page: Unitigs that significantly correlate with **norfloxacin** resistance (Bonferroni-corrected p-value threshold: 8.06×10^{-8}). The effect field shows any mutations in the unitigs that could have contributed to the resistance. If there is a C superscript in this field, it means the mutations occurred in the complementary set of isolates without this unitig, indicating a correlation with antibiotic susceptibility instead of resistance, as reflected by the sign of the effect size (beta). The frequency (freq.) field shows the relative frequency among isolates, while beta denotes the effect size, and SE denotes the standard error of the fit on beta.

Position	Length	Gene	Effect	Freq.	Beta	SE	P-value
3783537-3783576	40	*gpsA*	A42A^C	0.97	2.68	0.44	3.14×10^{-08}
3783516-3783566	51	*gpsA*	V32V^C	0.96	2.65	0.44	4.02×10^{-08}
1203247-1203292	46	*ymfL*	I8V<br>modifier	0.17	-1.30	0.21	3.15×10^{-08}
3777392-3777452	61	*lldP*	Y8Y<br>S18S	0.03	-2.68	0.44	3.14×10^{-08}
3777392-3777439	48	*lldP*	Y8Y^C<br>S18S^C	0.97	2.68	0.44	3.14×10^{-08}
3749279-3749317	39	*sgbU*	A26A	0.03	-2.68	0.44	3.14×10^{-08}
3744800-3744869	70	*yiaM* & *yiaN*	C9C<br>G13G	0.03	-2.68	0.44	3.14×10^{-08}
3744751-3744811	61	*yiaM* & *yiaN*	S152P	0.03	-2.68	0.44	3.14×10^{-08}
3744897-3744934	38	*yiaN*	D36D	0.03	-2.68	0.44	3.14×10^{-08}
3744642-3744683	42	*yiaM*	G115G<br>L116L	0.03	-2.68	0.44	3.14×10^{-08}
3744705-3744740	36	*yiaM*	E136E	0.03	-2.68	0.44	3.14×10^{-08}
3755421-3755457	37	*aldB*	A370A^C<br>G365G^C<br>I364I^C<br>N360N^C<br>L355L^C<br>V350V^C<br>G347G^C	0.96	2.65	0.44	4.02×10^{-08}
3810375-3810411	37	*mutM*	V252M^C	0.97	2.68	0.44	3.14×10^{-08}
3814136-3814168	33	*dut*	A65A^C<br>A66A^C<br>R71R^C	0.96	2.65	0.44	4.02×10^{-08}
1926954-1926985	32	*ptrB*	V629I^C	0.96	2.70	0.45	3.68×10^{-08}
883768-883820	53	*mdfA*	A42A^C	0.96	2.65	0.44	4.02×10^{-08}

Supplementary Table 3: Unitigs that significantly correlate with **ciprofloxacin** resistance (Bonferroni-corrected p-value threshold: 8.06×10^{-8}). The effect field shows any mutations in the unitigs that could have contributed to the resistance. If there is a C superscript in this field, it means the mutations occurred in the complementary set of isolates without this unitig, indicating a correlation with antibiotic susceptibility instead of resistance, as reflected by the sign of the effect size (beta). An E superscript in the effect field denotes the effect size for the extension of the unitig when the initial mapping attempt was unsuccessful. The frequency (freq.) field shows the relative frequency among isolates, while beta denotes the effect size, and SE denotes the standard error of the fit on beta.

Position	Length	Gene	Effect	Freq.	Beta	SE	P-value
3165735-3165765	31	*parC*	$S80I^{C}$	0.92	2.10	0.29	1.70×10^{-10}
3165705-3165753	49	*parC*	$S80I^{C}$	0.77	1.56	0.26	7.38×10^{-08}
3165724-3165761	38	*parC*	$S80I^{C}$	0.78	1.67	0.27	3.17×10^{-08}
2339132-2339192	61	*gyrA*	R91R<br>D87N<br>V85V<br>S83L	0.04	-2.25	0.37	4.54×10^{-08}
2096739-2096779	41	*hisI*	L46I	0.03	-2.84	0.41	6.97×10^{-10}
2096760-2096799	40	*hisI*	T52T	0.03	-2.84	0.41	6.97×10^{-10}
1429777-1429893	117	*stfR*	E244E<br>A253A<br>S254S<br>S255T<br>S258S<br>A261A<br>A271V	0.07	-1.98	0.31	5.34×10^{-09}
1429867-1429918	52	*stfR*	N280N<br>G289E	0.07	-1.98	0.31	5.34×10^{-09}
1429889-1429944	56	*stfR*	S293S	0.08	-1.75	0.29	5.64×10^{-08}
1203582-1203717	37	*ymfL*	$I107I^{E}$	0.09	-1.70	0.27	8.16×10^{-09}
1203582-1203712	32	*ymfL*	$S119S^{E}$	0.09	-1.70	0.27	8.16×10^{-09}
1429866-1429950	33	*stfR*	G289E	0.08	-1.73	0.29	8.00×10^{-08}

Supplementary Table 4: Unitigs that significantly correlate with **nalidixic acid** resistance (Bonferroni-corrected p-value threshold: 8.06×10^{-8}). The effect field shows any mutations in the unitigs that could have contributed to the resistance. If there is a C superscript in this field, it means the mutations occurred in the complementary set of isolates without this unitig, indicating a correlation with antibiotic susceptibility instead of resistance, as reflected by the sign of the effect size (beta). The frequency (freq.) field shows the relative frequency among isolates, while beta denotes the effect size, and SE denotes the standard error of the fit on beta.

Position	Length	Gene	Effect	Freq.	Beta	SE	P-value
2339167-2339203	37	*gyrA*	$S83L^{C}$	0.86	1.57	0.26	2.69×10^{-08}
